# Molecular and Translational Research on Bone Tumors

**DOI:** 10.3390/ijms24031946

**Published:** 2023-01-18

**Authors:** Michela Rossi, Andrea Del Fattore

**Affiliations:** Bone Physiopathology Research Unit, Translational Pediatrics and Clinical Genetics Research Division, Bambino Gesù Children’s Hospital, IRCCS, 00146 Rome, Italy

Primary bone tumors (PBTs) represent a huge variety of rare malignancies that originate in the skeletal system [1]. Although the whole skeleton may be affected by PBTs, the long bones are the most preferentially affected sites. Patients suffer from pain, swelling that impacts movements, and fractures. PTBs affect mainly pediatric subjects and young adults [2].

Benign PBTs may be classified into five categories, depending on the type of tissue the tumor has started from. The five categories are bone-forming tumors, cartilage-forming tumors, connective tissue tumors, vascular tissue tumors, and idiopathic tumors (giant cell tumor, aneurysmal bone cyst, and simple bone cyst) [3,4]. Among these categories, osteochondroma is the most prominent, accounting for 30–35% of total benign PBTs. The other less common benign tumors include enchondroma, aneurysmal bone cyst, periosteal chondroma, and osteoblastoma [5,6].

Despite accounting for only 0.2% of total tumors, malignant PBTs are extremely aggressive and difficult to treat; indeed, they are characterized by many histological variations, highlighting the problems related to the identification of resolutive therapies. About 80% of malignant PBTs are bone sarcomas (osteosarcoma, Ewing’s sarcoma, and chondrosarcoma) and are mainly treated with a combination of surgical strategies and/or chemotherapy [7].

The current status of therapies for bone sarcomas, as well as the problems correlated with the development of future therapies, are reviewed by Nakano et al. [8]. The authors analyzed how the progression of investigations on bone sarcomas’ therapies has been slower than the investigations on other tumors, due to the rarity of these bone malignancies. Moreover, Nakano and co-authors reported the problem related to the evaluation of different treatments’ efficacy because large-scale randomized clinical trials for osteosarcoma are lacking [8].

The most frequently observed bone sarcoma is osteosarcoma (OS) that occurs mainly in tubular bones following the defects of mesenchymal osteogenic differentiation [9,10,11]. The most widely used chemotherapeutic drugs for the treatment of OS are methotrexate, doxorubicin, and cisplatin, but consistent side effects and drug resistance often lead to treatment failure and poor prognosis. On this topic, Gerardo-Ramirez and co-authors analyzed the resistance to doxorubicin of osteosarcoma cells lacking CD44, and highlighted the role of this molecule as a drug resistance regulator and as a new target to improve drug sensitivity for osteosarcoma [12]. Moreover, in the original article by Wang et al., the estrogen receptor alpha was investigated as a possible new target to improve drug chemosensitivity [13].

Osteosarcoma has a high rate of recurrence and metastasis, with a 5-year survival rate of 27%. This poor survival rate indicates the need for new targets and new therapies for OS. Harris et al. reviewed the data obtained from clinical trials of therapies and from preclinical studies reporting the efficacy of 39 new drugs to treat metastatic osteosarcoma, including Eribulin, Tegavivint, Anlotinib, and Auranofin [14]. In the study by Mikulcic et al., a new natural compound, 15-Deoxy-D^12,14^-prostaglandin J2 (15d-PGJ_2_), was tested as an anti-tumoral drug, showing an anti-proliferative effect in different OS cell lines [15]. Furthermore, Mizerska-Kowalska and co-authors evaluated the effect of an organophosphorus compound derivative of α-aminophosphonate on OS, showing an anti-tumoral effect on highly aggressive HOS cell lines [16].

New technologies, such as proteomic, transcriptomic, and metabolomic analyses, are powerful tools to investigate the molecular mechanisms underlying the development and growth of osteosarcoma. In the research study by Madda et al., a targeted proteomic technology was used to investigate the protein expression of components of the growth factor families (BMP2, bone morphogenetic protein 2; FGFR, fibroblast growth factor receptor; and TGF-beta, transforming growth factor beta) with a fundamental role in bone remodeling and regeneration from treated and untreated autografts [17].

OS cells interact with the microenvironment via the secretion and/or uptake of extracellular vesicles (EVs), which are small double-layered particles that transport important signaling molecules [18]. Recent papers have highlighted the significant role of EVs in OS development, progression, and aggressiveness, pointing out the use of EVs as tumoral biomarkers, both for prognosis and diagnosis; these papers have been well reviewed by De Martino et al. [19]. Moreover, Luu and co-authors developed a new pipeline to isolate and characterize EVs directly from tumoral tissue, identifying a typical proteomic signature for tumor [20].

This Special Issue of the *International Journal of Molecular Sciences*, with two reviews and seven original research articles, aims to report and summarize current molecular and translational research on bone tumors, providing novel insights into the mechanisms associated with cancer progression and metastasis.

We are very grateful to all the authors who submitted a manuscript and contributed to the publication of this successful Special Issue.
